# MOBILIZING COMMUNITY PARTNERSHIPS TO ENHANCE HEALTH AND REDUCE INEQUITIES IN MULTICULTURAL COMMUNITIES

**DOI:** 10.1093/geroni/igz038.2733

**Published:** 2019-11-08

**Authors:** Daniel S Gardner, Nancy Giunta

**Affiliations:** Silberman School of Social Work, Hunter College, CUNY, New York, New York, United States

## Abstract

Community-based gerontological research plays an indispensable role in identifying and addressing the strengths, intersectionalities, and socio-structural inequities that shape the lives of older adults in multicultural communities around the world. This symposium highlights the innovative, global scholarship of Silberman Aging: A Hartford Center of Excellence in Diverse Aging, as the Center begins its sixth year. Through community-based research and academic-community collaborations, Center researchers examine challenges affecting the health and wellbeing of diverse and often marginalized aging communities in North America, West Africa, and East Asia. The first paper describes and evaluates a CBPR project that trains community-based natural helping networks to identify and refer older adults with dementia in East Harlem, NY. The second study explores the perceptions and strategies of community-based primary care physicians in Ulaanbaatar, Mongolia in dealing with elder abuse and neglect. The third takes a population health approach to the relationship between social capital and health among older adults in Ghana. Fourth, preliminary results from an evaluation of a nation-wide training initiative that promotes cultural-competencies among aging services providers working with LGBT elders. Finally, we present findings from a CBPR study examining barriers to palliative care among racially and ethnically-diverse community-dwelling older adults with serious illness. Although substantively and methodologically varied, these studies all demonstrate the importance of social networks in health in later life, and underscore the value of community-based research that supports collaboration, empowers communities, and ultimately transforms practice and policy to better meet the diverse needs of older adults around the globe.

